# Temporal changes in dilated ascending aorta after transfemoral TAVR: a comparative analysis of bicuspid and tricuspid aortic valves

**DOI:** 10.3389/fcvm.2026.1878963

**Published:** 2026-10-09

**Authors:** Qichen Xu, Zheng Xu, Xiaofu Dai, Liangwan Chen, Zhiqin Lin

**Affiliations:** 1 Department of Cardiovascular Surgery, Fujian Provincial Center for Cardiovascular Medicine, Union Hospital, Fujian Medical University, Fuzhou, China; 2 Key Laboratory of Cardio-Thoracic Surgery, Fujian Medical University, Fuzhou, Fujian, China

**Keywords:** aortic remodeling, aortopathy, ascending aortic dilatation, bicuspid aortic valve, transcatheter aortic valve replacement

## Abstract

**Background:**

Transcatheter aortic valve replacement (TAVR) addresses valvular obstruction but leaves concomitant ascending aortic dilatation untreated. Whether bicuspid aortic valve (BAV) morphology inherently accelerates post-TAVR aortic expansion compared with tricuspid aortic valves (TAV) remains uncertain.

**Methods:**

This retrospective cohort study included 189 older adults (BAV, *n* = 87; TAV, *n* = 102) with severe aortic stenosis and ascending aortic dilatation (≥4.5 cm) undergoing TAVR. Annualized aortic expansion rates were evaluated utilizing linear mixed-effects models derived from serial computed tomography angiography over a median 27.0-month follow-up.

**Results:**

No ascending aortic dissection, rupture, or elective aortic interventions occurred during follow-up. The adjusted annualized expansion rate at the mid-ascending aorta was low and comparable between the BAV and TAV cohorts (0.43 vs. 0.39 mm/year; interaction *P* = 0.364). Progression rates at the distal ascending aorta and sinuses of Valsalva similarly demonstrated no significant morphological differences. No patient exhibited rapid progression (>5 mm/year). Although moderate or greater paravalvular leakage occurred exclusively in BAV patients (4.8% vs. 0.0%; *P* = 0.043), its overall incidence was low (2.2%); however, the extremely small number of cases precluded a definitive statistical assessment of its impact on longitudinal aortic expansion.

**Conclusions:**

In this selected cohort, dilated ascending aortas exhibited slow intermediate-term expansion following transfemoral TAVR utilizing self-expanding valves, with comparable rates between BAV and TAV morphologies. These findings suggest that conservative management of concomitant ascending aortic dilatation may be a reasonable strategy in older TAVR candidates during intermediate-term follow-up, provided that strict blood pressure control and rigorous imaging surveillance are maintained.

## 1 Introduction

Aortic stenosis (AS) is the most prevalent valvular heart disease among older adults and is frequently associated with ascending aortic dilatation (1). This coexistence poses a significant therapeutic challenge, particularly as indications for transcatheter aortic valve replacement (TAVR) expand to younger, lower-risk patients who would traditionally undergo surgical aortic valve replacement (SAVR) (2). Current surgical guidelines recommend concomitant replacement of the ascending aorta when the diameter exceeds 4.5–5.0 cm to mitigate late complications such as dissection or rupture (3). However, TAVR resolves only the valvular obstruction, leaving the dilated aorta untreated. Consequently, the natural history of this residual aortic pathology remains poorly understood, raising concerns regarding the long-term safety of omitting concurrent aortic intervention (4).

Aortic valve morphology remains a critical yet understudied determinant of post-TAVR aortic remodeling. Theoretically, the severe flow turbulence and high-velocity eccentric jets associated with AS exert elevated wall shear stress, thereby promoting post-stenotic dilatation (5). Accordingly, alleviating valvular stenosis via TAVR may improve transvalvular hemodynamics, mitigate the impact of eccentric flow on the ascending aortic wall, and consequently attenuate further aortic enlargement (6). However, this hypothesis is challenged by the presence of intrinsic genetic aortopathies, particularly in patients with bicuspid aortic valve (BAV) morphology (7). If the aortic wall is structurally compromised by medial degeneration, dilatation may persist even after hemodynamic stressors are alleviated (8). This uncertainty is particularly relevant for older adults with moderate-to-severe ascending aortic dilatation. Although many of these patients present a low-to-intermediate risk for isolated surgical aortic valve replacement, they are frequently deemed suboptimal candidates for concomitant aortic replacement due to the substantial procedural morbidity and extended recovery associated with extensive open-heart surgery.

Current evidence on post-TAVR progression of aortic dilatation remains limited and is largely derived from heterogeneous or predominantly tricuspid aortic valve populations. To address this knowledge gap, we conducted a retrospective study in patients with severe AS and concomitant ascending aortic dilatation undergoing transfemoral TAVR. The primary objective was to characterize the mid-term trajectory of aortic dimensions, comparing annualized expansion rates and adverse events between BAV and tricuspid aortic valve (TAV) cohorts. We aimed to determine whether post-TAVR aortic progression differs between these morphological phenotypes and whether relief of valvular obstruction is associated with stabilization of ascending aortic dimensions in both groups.

## 2 Methods

### 2.1 Study design and population

We conducted a single-center, retrospective cohort study of a selected cohort of patients with severe symptomatic AS and concomitant ascending aortic dilatation who underwent transfemoral TAVR at our institution between January 2019 and November 2025. Inclusion criteria included adults aged ≥65 years with severe AS and ascending aortic dilatation, defined as a diameter of ≥4.5 cm at the mid- or distal ascending aortic level on pre-procedural computed tomography angiography (CTA). These patients were deemed at high or prohibitive surgical risk for combined open surgery by the institutional Heart Team due to advanced age and comorbidities. Specifically, TAVR was selected for these patients due to the prohibitive physiological burden of combined SAVR and ascending aortic replacement (often requiring circulatory arrest), unmeasured clinical frailty, specific anatomical challenges (e.g., porcelain aorta, hostile chest), or explicit patient refusal of open-heart surgery following a comprehensive shared decision-making process. Only patients for whom the Heart Team explicitly advised against prophylactic aortic intervention were included. Severe AS was defined according to contemporary guideline criteria, which included an aortic valve area ≤1.0 cm^2^, a mean transvalvular gradient ≥40 mmHg, or a peak aortic jet velocity ≥4.0 m/s; patients with low-flow, low-gradient AS were included only if the diagnosis of true severe AS was confirmed via an integrated clinical and imaging assessment. Exclusion criteria encompassed isolated aortic regurgitation, non-transfemoral access (e.g., transapical or transaortic), prior aortic valve or ascending aortic surgery, intraoperative conversion to open-heart surgery, known connective tissue disorders (e.g., Marfan syndrome), active aortitis, and incomplete clinical or imaging data that precluded reliable assessment. For comparative analysis, patients were stratified into BAV and TAV cohorts based on their pre-procedural CTA morphology. The study protocol was approved by the Ethics Committee of Union Hospital, Fujian Medical University, and the requirement for informed consent was waived due to the retrospective study design.

### 2.2 Data collection and outcome measures

Clinical data, including demographics, comorbidities, procedural details, imaging findings, and follow-up outcomes, were retrospectively extracted from electronic health records. Baseline aortic dimensions were obtained from pre-procedural CTA. Follow-up CTA was performed at 3 months, 1 year, and annually thereafter, with a data cutoff date of February 20, 2026. CTA images were analyzed using multiplanar reconstructions on dedicated software (3mensio Structural Heart, Pie Medical Imaging, Maastricht, the Netherlands). To ensure reproducibility, aortic diameters were measured perpendicular to the aortic centerline on ECG-gated images reconstructed at end-diastole (70%–75% of the R-R interval). All baseline and follow-up CTA studies were strictly ECG-gated and acquired using a standardized institutional protocol uniformly applied across the entire study period. Measurements were obtained at three predefined levels: (1) the mid-ascending aorta (tubular portion between the sinotubular junction and pulmonary artery bifurcation), (2) the distal ascending aorta (immediately proximal to the innominate artery), and (3) the sinuses of Valsalva (Figures 1A,B). The mid-ascending aorta was measured at the site of maximal diameter referenced to the aortic annular plane. All measurements were independently performed by two experienced cardiovascular radiologists blinded to clinical data. Inter-observer variability was assessed using the intraclass correlation coefficient, with discrepancies >2 mm resolved by consensus.

**Figure 1:**
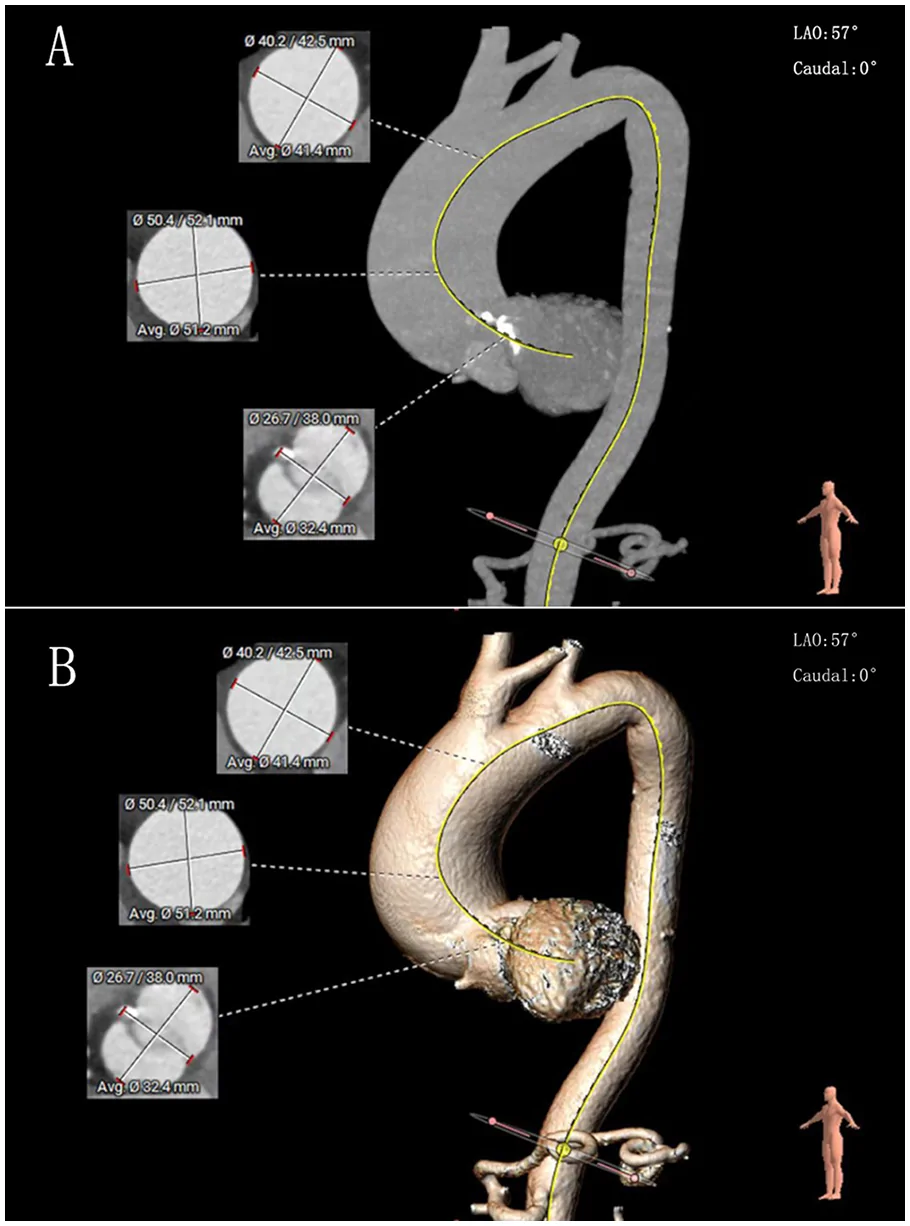
Measurement of the ascending aorta utilizing computed tomography angiography (CTA). **(A)** A centerline-based CTA overview image demonstrating the planned measurement levels along the ascending aorta. To ensure accuracy and reproducibility, all true quantitative diameter measurements were performed exclusively on cross-sectional multiplanar reconstruction (MPR) images strictly perpendicular to the central axis of the aorta. **(B)** A three-dimensional (3D) volume-rendered reconstruction illustrating the anatomical morphology of the dilated ascending aorta and the relationship of the predefined measurement landmarks: the sinuses of Valsalva, the mid-ascending aorta, and the distal ascending aorta.

The primary outcome was the annualized rate of change in mid-ascending aortic diameter (mm/year), calculated using linear mixed-effects models on serial CTA measurements. This level was chosen as it is most susceptible to post-stenotic dilatation and corresponds to established surgical intervention thresholds. Secondary outcomes included annualized expansion rates at the distal ascending aorta and sinuses of Valsalva, major aortic events (dissection, rupture, or urgent intervention), significant radiographic progression (>10 mm increase), rapid progression (defined primarily as an annualized expansion rate ≥5 mm/year, corresponding to the ACC/AHA guideline threshold for surgical intervention, with a stricter sensitivity threshold set at ≥3 mm/year for enhanced detection of subclinical rapid growth), overall survival, freedom from aortic reintervention, and aortic-related mortality. The primary comparison was the difference in temporal aortic diameter trajectories between BAV and TAV groups using the time-by-group interaction term.

### 2.3 TAVR procedure and postprocedural management

All TAVR procedures were performed via the transfemoral approach under conscious sedation or general anesthesia, as determined by the institutional Heart Team. Self-expanding transcatheter heart valves were predominantly utilized, including the Venus A-Plus (Venus MedTech, Hangzhou, China), TaurusElite (Peijia Medical, Suzhou, China), and VitaFlow Liberty (MicroPort Scientific, Shanghai, China), all of which feature a supra-annular leaflet design. Prosthesis sizing was determined using CTA-derived annular dimensions. For BAV cases, supra-annular structures and the inter-commissural distance were further evaluated to minimize the risk of paravalvular leakage and annular injury. Because all patients exhibited ascending aortic dilatation, catheter manipulation was executed cautiously to prevent aortic wall trauma. Intraprocedural angiography was conducted to confirm coronary patency and evaluate paravalvular regurgitation. Strict perioperative blood pressure control was maintained during the index hospitalization with a target systolic blood pressure of <120 mmHg, adhering to contemporary aortic disease management guidelines. Upon discharge, patients were counseled to maintain this target, although subsequent long-term antihypertensive management was directed by primary care providers. Postprocedural antithrombotic therapy followed institutional protocols, comprising dual antiplatelet therapy (100 mg of aspirin plus 75 mg of clopidogrel daily) for 3–6 months, followed by aspirin monotherapy unless oral anticoagulation was indicated.

### 2.4 Statistical analysis

Continuous variables are presented as mean ± standard deviation or median (interquartile range) according to normality and compared using Student's *t*-test or Mann–Whitney *U* test, as appropriate. Categorical variables are expressed as frequencies (percentages) and compared using chi-square or Fisher's exact test. Temporal aortic diameter changes were analyzed using linear mixed-effects models with random intercepts and random slopes for time. Fixed effects included time (in months post-TAVR), valve morphology (BAV vs. TAV), and the time-by-group interaction term. Models were adjusted for age, sex, hypertension, and baseline aortic regurgitation severity. Slope coefficients were multiplied by 12 to obtain annualized expansion rates (mm/year). Missing CTA data were assumed to be missing at random, and no imputation was performed. Model assumptions were checked using residual and Q-Q plots. Time-to-event outcomes were analyzed using Kaplan–Meier curves and the log-rank test. A two-sided *P* value <0.05 was considered statistically significant. Analyses were performed utilizing SPSS version 26.0 (IBM Corp, Armonk, NY) and R version 4.4.0 (R Foundation for Statistical Computing, Vienna, Austria).

## 3 Results

### 3.1 Patient population and baseline characteristics

To provide institutional context, during the study period (January 2019 to November 2025), our center performed a total of 1,236 TAVR procedures (averaging approximately 180 cases per year), with an overall institutional BAV prevalence of 32.0% (395/1,236). From this broader population, a total of 189 consecutive patients with severe symptomatic AS and ascending aortic dilatation were included and stratified into BAV (*n* = 87) and TAV (*n* = 102) cohorts. Baseline characteristics are summarized in Table 1. Patients in the BAV cohort were slightly younger than those in the TAV cohort (median age 72 vs. 74 years; *P* = 0.003), although this difference was clinically modest. The estimated surgical risk was low to intermediate in both groups (median EuroSCORE II 2.45% vs. 3.58%; *P* = 0.504). Sex distribution was comparable (56.3% vs. 49.0% male; *P* = 0.316). Hypertension was more common in the TAV cohort (36.3% vs. 19.5%; *P* = 0.011), while moderate or greater aortic regurgitation was more frequent in the BAV cohort (42.5% vs. 22.5%; *P* = 0.003). Other comorbidities, New York Heart Association functional class, and baseline echocardiographic parameters did not differ significantly between the cohorts. All aortic measurements were independently performed by two experienced cardiovascular radiologists blinded to clinical data, and the inter-observer variability assessment demonstrated excellent agreement, with an intraclass correlation coefficient (ICC) of 0.994 (95% CI: 0.984–0.998). At baseline, the exact distribution of mid-ascending aortic diameters across the entire cohort was as follows: 86 patients (45.5%) had a diameter of 45.0–49.9 mm, 61 patients (32.3%) had 50.0–54.9 mm, and 42 patients (22.2%) had ≥55.0 mm. Furthermore, the baseline aortic dimensions indexed to body surface area were comparable between the BAV and TAV cohorts (median 2.2 vs. 2.2 mm/m^2^; *P* = 0.657). Within the BAV cohort (*n* = 87), morphological subclassification according to the Sievers system revealed that Type 0 was the most prevalent morphology, observed in 48 patients (55.2%), followed by Type 1 in 38 patients (43.7%), and Type 2 in 1 patient (1.1%). Regarding the aortopathy phenotype within the BAV cohort, the ascending phenotype was universally present (observed in 87 patients, 100.0%), with no patients exhibiting a root-predominant phenotype.

**Table 1:** Comparison of patients’ baseline demographic and clinical characteristics.

Variables^a^	Total sample (*n* = 189)	Patient groups		*P* value
		BAV (*n* = 87)	TAV (*n* = 102)	
Age, years	72 [71, 76]	72 [70, 74]	74 [71, 77]	0.003
Male, *n* (%)	99 (52.4%)	49 (56.3%)	50 (49.0%)	0.316
BMI, kg/m^2^	23.4 [21.0, 25.1]	23.5 [21.6, 25.4]	23.2 [20.6, 24.9]	0.168
Smoking history, *n* (%)	42 (22.2%)	21 (24.1%)	21 (20.6%)	0.559
Comorbidities				
Diabetes, *n* (%)	67 (35.4%)	30 (34.5%)	37 (36.3%)	0.797
Hypertension, *n* (%)	54 (28.6%)	17 (19.5%)	37 (36.3%)	0.011
COPD, *n* (%)	47 (24.9%)	19 (21.8%)	28 (27.5%)	0.374
CAD, *n* (%)	59 (31.2%)	28 (32.2%)	31 (30.4%)	0.791
Previous myocardial infarction, *n* (%)	33 (17.5%)	14 (16.1%)	19 (18.6%)	0.647
Stroke history, *n* (%)	29 (15.3%)	10 (11.5%)	19 (18.6%)	0.175
Liver dysfunction, *n* (%)	32 (16.9%)	10 (11.5%)	22 (21.6%)	0.066
Dialysis, *n* (%)	22 (11.6%)	8 (9.2%)	14 (13.7%)	0.333
Peripheral vascular disease, *n* (%)	22 (11.6%)	8 (9.2%)	14 (13.7%)	0.333
Cancer history, *n* (%)	27 (14.3%)	11 (12.6%)	16 (15.7%)	0.551
AF, *n* (%)	48 (25.4%)	21 (24.1%)	27 (26.5%)	0.713
NYHA, *n* (%)				0.140
NYHA I	0 (0%)	0 (0%)	0 (0%)	
NYHA II	25 (13.2%)	9 (10.3%)	16 (15.7%)	
NYHA III	129 (68.3%)	57 (65.5%)	72 (70.6%)	
NYHA IV	35 (18.5%)	21 (24.1%)	14 (13.7%)	
Prior cardiac surgery, *n* (%)	25 (13.2%)	10 (11.5%)	15 (14.7%)	0.516
EuroSCORE II score, %	2.86 [1.67, 6.66]	2.45 [1.63, 6.58]	3.58 [1.72, 6.80]	0.504
Baseline ascending aortic dimensions				
Size distribution, *n* (%)				0.143
45.0–49.9 mm	86 (45.5%)	33 (37.9%)	53 (52.0%)	
50.0–54.9 mm	61 (32.3%)	33 (37.9%)	28 (27.5%)	
≥55.0 mm	42 (22.2%)	21 (24.1%)	21 (20.6%)	
BSA-indexed diameter, mm/m^2^	2.2 [2.0, 2.4]	2.2 [2.0, 2.4]	2.2 [2.0, 2.4]	0.657
Echocardiographic parameters				
Moderate or severe aortic valve regurgitation, *n* (%)	60 (31.7%)	37 (42.5%)	23 (22.5%)	0.003
Mean transaortic pressure gradient, mmHg	52.7 [50.9, 55.1]	52.8 [51.3, 55.3]	52.7 [49.4, 55.0]	0.209
Moderate or severe mitral regurgitation, *n* (%)	19 (10.1%)	9 (10.3%)	10 (9.8%)	0.902
LVEF, %	55.8 [46.4, 60.6]	55.1 [45.0, 60.1]	56.9 [48.4, 61.1]	0.099

BAV, bicuspid aortic valve; TAV, tricuspid aortic valve; BMI, body mass index; COPD, chronic obstructive pulmonary disease; CAD, coronary artery disease; AF, atrial fibrillation; NYHA, New York Heart Association; LVEF, left ventricular ejection fraction.

a Normally distributed variables are presented as mean ± standard deviation, non-normally distributed variables are presented as median [interquartile range (IQR)], and categorical variables are presented as number (%).

### 3.2 Procedural details and in-hospital outcomes

All procedures were performed via the transfemoral approach. Procedural details and early clinical outcomes are summarized in Table 2. Balloon pre-dilation (93.1% vs. 82.4%, *P* = 0.027) and post-dilation (44.8% vs. 25.5%, *P* = 0.005) were performed significantly more often in the BAV cohort than in the TAV cohort. The need for an intraprocedural second valve (bailout valve-in-valve) due to valve malposition or severe acute regurgitation was comparable between the BAV and TAV cohorts (5.7% vs. 2.0%, *P* = 0.323). Overall in-hospital mortality was 2.1% (4/189), with no significant difference between the cohorts (2.3% vs. 2.0%, *P* = 0.729). Neurological complications were numerically more frequent in the TAV cohort (2.9% vs. 1.1%), although this difference was not statistically significant (*P* = 0.114). The incidence of new-onset dialysis was comparable between the cohorts (1.1% vs. 3.9%, *P* = 0.466). Other complications, including perioperative myocardial infarction (1.1% vs. 2.0%, *P* > 0.999) and pulmonary events (4.6% vs. 2.0%, *P* = 0.539), were similar between the cohorts. The need for mechanical circulatory support, specifically intra-aortic balloon pump or extracorporeal membrane oxygenation, was exclusively observed in the TAV cohort (2.0% for each), but this difference lacked statistical significance.

**Table 2:** Operative details and in-hospital postoperative outcomes.

Variables^a^	Total sample (*n* = 189)	Patient groups		*P* value
		BAV (*n* = 87)	TAV (*n* = 102)	
Operative details				
Balloon pre-dilation, *n* (%)	165 (87.3%)	81 (93.1%)	84 (82.4%)	0.027
Balloon post-dilation, *n* (%)	65 (34.4%)	39 (44.8%)	26 (25.5%)	0.005
Second valve implantation, *n* (%)	7 (3.7%)	5 (5.7%)	2 (2.0%)	0.323
Post-TAVR mean gradient, mmHg	11.3 ± 3.7	12.5 ± 3.6	10.4 ± 3.5	<0.001
Hospital mortality, *n* (%)	4 (2.1%)	2 (2.3%)	2 (2.0%)	>0.999
Early postoperative outcomes				
Pulmonary events^b^, *n* (%)	6 (3.2%)	4 (4.6%)	2 (2.0%)	0.539
Postoperative myocardial infarction, *n* (%)	3 (1.6%)	1 (1.1%)	2 (2.0%)	>0.999
Cardiocerebral events, *n* (%)	4 (2.1%)	1 (1.1%)	3 (2.9%)	0.729
New-onset dialysis, *n* (%)	5 (2.6%)	1 (1.1%)	4 (3.9%)	0.466
IABP, *n* (%)	2 (1.1%)	0 (0.0%)	2 (2.0%)	0.501
ECMO, *n* (%)	2 (1.1%)	0 (0.0%)	2 (2.0%)	0.501
Late clinical outcomes^c^				
Death, *n* (%)	18 (9.7%)	7 (8.2%)	11 (11.0%)	0.527
PVL^d^, *n* (%)				0.090
None or trace	172 (95.6%)	77 (92.8%)	95 (97.9%)	
Mild	4 (2.2%)	2 (2.4%)	2 (2.1%)	
Moderate or more	4 (2.2%)	4 (4.8%)	0 (0.0%)	

BAV, bicuspid aortic valve; TAV, tricuspid aortic valve; TAVR, transcatheter aortic valve replacement; IABP, intra-aortic balloon pump; ECMO, extracorporeal membrane oxygenation; PVL, paravalvular leakage.

a Normally distributed variables are presented as mean ± standard deviation, non-normally distributed variables are presented as median [interquartile range (IQR)], and categorical variables are presented as number (%).

b Pulmonary events were defined as postoperative pneumonia, acute respiratory distress syndrome, prolonged mechanical ventilation (>48 h), or the need for reintubation.

c Late clinical outcomes represent events during the follow-up period for the 185 patients who survived to discharge.

d PVL data represent the latest available echocardiographic assessment for surviving patients. The analysis includes 180 patients with evaluable imaging (BAV: *n* = 83; TAV: *n* = 97); patients who died prior to follow-up imaging or had missing echocardiographic data were excluded from this specific analysis.

### 3.3 Intermediate-term survival, echocardiographic follow-up, and major aortic events

Of the 189 patients included, 4 (2.1%) died during the index hospitalization. The remaining 185 patients were followed for a median of 27.0 months (IQR: 11.0–50.0). Kaplan–Meier analysis demonstrated an overall survival of 95.7% at 1 year and 87.3% at 3 years, with no significant difference between the BAV and TAV cohorts (89.6% vs. 85.1% at 3 years, log-rank *P* = 0.670). Among the 180 patients with evaluable follow-up echocardiography, the overall incidence of moderate or greater PVL was 2.2%. Moderate or greater PVL occurred exclusively in the BAV cohort (4.8% vs. 0.0% in the TAV cohort, *P* = 0.043), whereas mild PVL rates were comparable between the cohorts (2.4% vs. 2.1%, overall *P* = 0.090). All cases of moderate PVL were managed conservatively with medical therapy and close surveillance. Importantly, given the very small absolute number of patients presenting with moderate or greater PVL (*n* = 4), the true impact of significant residual regurgitation on late longitudinal aortic expansion trajectories could not be definitively evaluated in this cohort. No aortic-related mortality, ascending aortic dissection, rupture, or elective ascending aortic intervention occurred during the follow-up period. One patient in the BAV cohort underwent thoracic endovascular aortic repair for a type B aortic dissection at an external facility; this event was not classified as an ascending aortic complication.

### 3.4 Time-course analysis of aortic progression

A time-course analysis was conducted using all available CTA scans from the study cohort, resulting in 785 observations over a median follow-up of 27 months (interquartile range: 11–50). Of the 185 patients who survived their initial hospitalization, 180 (97.3%) had both a baseline scan and at least one postoperative CTA available for review. Among these 180 patients, 38 had a total of two CTA scans (a baseline and one follow-up), 34 had three scans, and 108 had four or more scans. Comparing the baseline characteristics of the 180 included patients with the five patients excluded due to missing follow-up imaging revealed no significant differences in age, sex, EuroSCORE II, or baseline aortic dimensions (all *P* > 0.05). This supports the statistical assumption that the absence of follow-up imaging occurred at random. Baseline measurements indicated that patients in the BAV cohort had significantly larger dimensions at the sinuses of Valsalva compared with the TAV cohort (median, 40.7 vs. 39.7 mm; *P* = 0.034), whereas dimensions at the mid-ascending aorta (51.4 vs. 49.6 mm; *P* = 0.241) and distal ascending aorta (41.7 vs. 41.4 mm; *P* = 0.171) were comparable. Linear mixed-effects modeling, adjusted for age, sex, hypertension, and aortic regurgitation, was utilized to evaluate aortic progression. This analysis demonstrated a small but statistically significant increase in aortic diameter over time in the overall cohort (time effect, *P* < 0.001), although absolute annual growth rates remained low across all anatomical levels. The adjusted annualized expansion rate of the mid-ascending aorta did not differ significantly between the BAV and TAV cohorts (0.43 [95% CI: 0.37–0.50] vs. 0.39 [95% CI: 0.33–0.45] mm/year; estimated between-group difference, 0.04 mm/year [95% CI: −0.05 to 0.13]; interaction *P* = 0.364). To address the distinct clinical implications of varying degrees of baseline dilatation, a sensitivity analysis stratified by a 50.0-mm threshold was conducted. The adjusted annualized expansion rate at the mid-ascending aorta was statistically significantly higher in patients with baseline diameters ≥50.0 mm compared to those with diameters 45.0–49.9 mm (0.46 mm/year vs. 0.35 mm/year; interaction *P* = 0.020). However, the absolute rate of expansion in the larger cohort remained clinically indolent (<0.5 mm/year). Similarly, progression rates at the distal ascending aorta (0.19 [0.12–0.25] vs. 0.23 [0.17–0.29] mm/year; estimated between-group difference, −0.04 mm/year [95% CI: −0.13 to 0.05]; interaction *P* = 0.370) and the sinuses of Valsalva (0.27 [0.20–0.33] vs. 0.24 [0.19–0.30] mm/year; estimated between-group difference, 0.02 mm/year [95% CI: −0.06 to 0.11]; interaction *P* = 0.577) did not differ significantly between the cohorts (Figures 2A–C). Although a small increase from baseline was observed within both cohorts over time (all *P* < 0.001), the longitudinal trajectories were not significantly different between the BAV and TAV morphologies. Categorical analyses further supported the relative stability of aortic dimensions during the follow-up period. Among the 180 patients with both baseline and at least one postoperative CTA available for assessment, only 16 (8.9%) showed an apparent absolute increase in aortic diameter >3 mm at any measured level. Importantly, no patients (0/180) exhibited significant radiographic progression (an absolute increase >10 mm). Furthermore, no patients (0/180) met the primary criterion for rapid progression (an annualized expansion rate ≥5 mm/year), and a sensitivity analysis confirmed that zero patients (0/180) exhibited an expansion rate of ≥3 mm/year (Figure 3).

**Figure 2:**
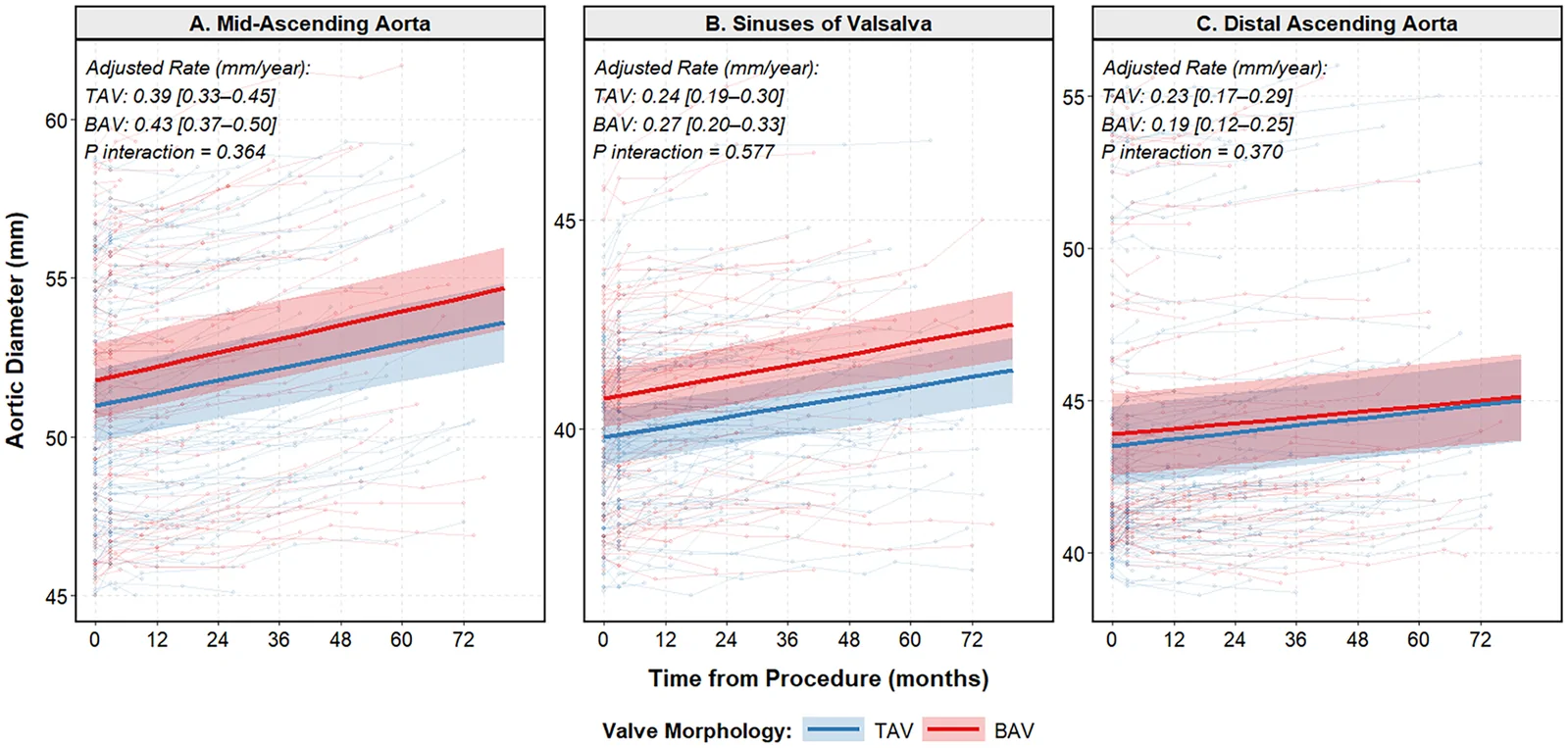
Longitudinal trajectories of aortic dimensions after transfemoral TAVR, stratified by valve morphology. Panels show the post-procedural progression of the mid-ascending aorta **(A)**, sinuses of Valsalva **(B)**, and distal ascending aorta **(C)** Faint lines and scatter points represent individual patient trajectories and CTA measurements. Solid lines and shaded ribbons denote the marginal predicted mean trajectories and 95% CIs for the BAV (red) and TAV (blue) cohorts. Progression rates and interaction *P*-values were estimated using a linear mixed-effects model adjusted for age, sex, hypertension, and baseline aortic regurgitation. BAV, bicuspid aortic valve; CI, confidence interval; CTA, computed tomography angiography; TAV, tricuspid aortic valve; TAVR, transcatheter aortic valve replacement.

**Figure 3:**
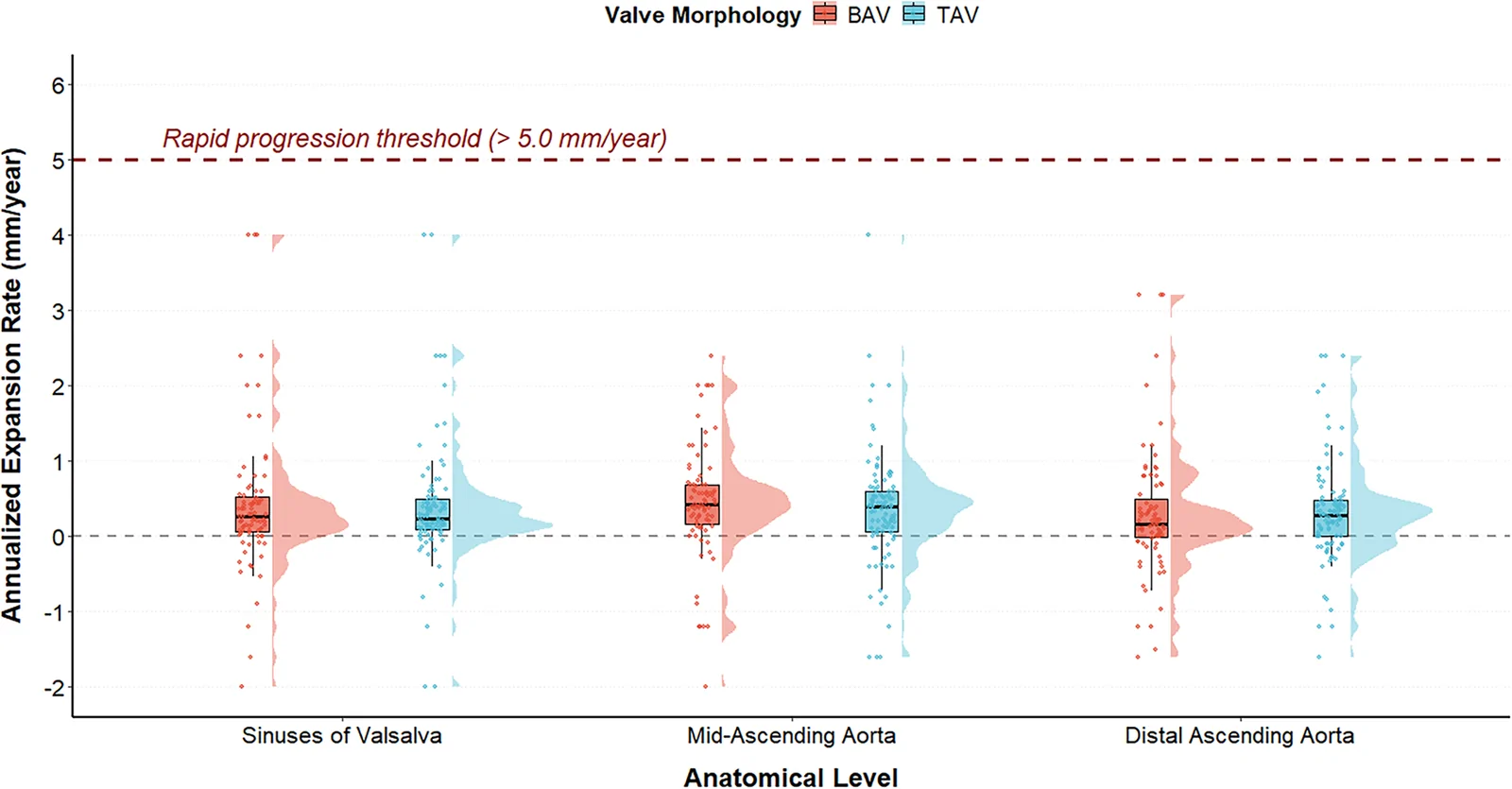
Distribution of individual annualized aortic expansion rates stratified by valve morphology. A grouped raincloud plot illustrates patient-level expansion rates across three anatomical levels (the sinuses of Valsalva, mid-ascending aorta, and distal ascending aorta), stratified by BAV (red) and TAV (blue) morphology. Jittered points represent individual rates calculated from baseline to the latest CTA. Box plots and half-violins display the median [IQR] and probability density, respectively. The gray dashed line indicates dimensional stability (0.0 mm/year), while the dark red dashed line marks the clinical threshold for rapid progression (>5.0 mm/year), which corresponds to the 2022 ACC/AHA guideline threshold for prophylactic surgical intervention. BAV, bicuspid aortic valve; CTA, computed tomography angiography; IQR, interquartile range; TAV, tricuspid aortic valve.

## 4 Discussion

This study demonstrates that in older adults with severe aortic stenosis and concomitant ascending aortic dilatation (≥4.5 cm) undergoing transfemoral TAVR, the ascending aorta exhibited slow expansion during intermediate-term follow-up, with comparable trajectories between bicuspid and tricuspid aortic valve morphologies. These findings demonstrate that post-TAVR aortic enlargement remains indolent in this selected population, irrespective of baseline valve morphology. Notably, despite baseline diameters approaching or exceeding conventional surgical thresholds, no ascending aortic dissection, rupture, or elective aortic intervention occurred during a median 27-month follow-up. These results support a strategy of conservative aortic management with close imaging surveillance in selected older adults at elevated surgical risk, although longer-term data remain necessary.

Our findings contribute to the literature by providing insights into an older TAVR population, which differs substantially from historical surgical cohorts. Current ACC/AHA guidelines recommend concomitant aortic replacement during SAVR for diameters >4.5 cm in high-risk BAV patients, or >5.0 cm generally. However, applying these surgical thresholds to older TAVR candidates is challenging, as the physiological burden of combined open-heart surgery often outweighs its prophylactic benefits (3). Previous surgical series, primarily involving younger and lower-risk patients, have suggested that isolated SAVR may not fully arrest aortic expansion in those with pre-existing dilatation. Matsuyama et al. reported that patients with a dilated ascending aorta (≥40 mm) at the time of SAVR remained at an elevated risk of late aortic events, even when baseline diameters were below conventional surgical thresholds (<50 mm) (8). Similarly, another surgical series with a 7.6-year follow-up identified an ascending aortic diameter of ≥40 mm at the time of SAVR as the sole independent predictor of late aortic complications (9).

However, direct extrapolation of these findings to contemporary TAVR populations remains challenging. First, historical surgical cohorts were substantially younger (mean age approximately 60 years) with longer life expectancy, allowing more time for slow aortopathic processes to manifest clinically (10). Second, SAVR involves cardiopulmonary bypass and direct aortic manipulation, which may trigger inflammatory remodeling distinct from the minimally invasive TAVR approach (11, 12). Third, competing risks of non-aortic mortality can mask late aortic events in traditional, prohibitive-risk TAVR cohorts. However, our study population presents a distinct clinical profile. With a median age of 72–74 years, these patients are relatively younger and possess a longer life expectancy than historical TAVR populations. Consequently, while our observed expansion rates of 0.39–0.43 mm/year indicate clinically acceptable intermediate-term stabilization, they do not guarantee long-term aortic safety. Given the extended life expectancy of this cohort, continuous and rigorous long-term imaging surveillance remains imperative.

In this context, our calculated expansion rates (0.39–0.43 mm/year) in an older adult TAVR cohort suggest a more benign natural history. These rates remain well below the threshold for rapid progression (≥5 mm/year) established by the 2022 ACC/AHA guidelines for the diagnosis and management of aortic disease (3). These findings align with prior TAVR studies demonstrating minimal, morphology-independent aortic growth during short- to intermediate-term follow-up (13, 14). This observation is further supported by Feng et al., who reported that moderate-to-severe (≥45 mm) compared with none-to-mild ascending aortic dilatation neither compromised 4-year survival nor increased late aortic events post-TAVR (15). Notably, aortic expansion in the dilated cohort was negligible and comparable to that of the none-to-mild group (0.0 [IQR: −0.3 to 0.2] vs. 0.1 [IQR: −0.3 to 0.4] mm/year; *P* = 0.122). Collectively, these studies indicate that dilated ascending aortas generally exhibit slow post-procedural expansion and low rates of clinically overt aortic events following TAVR, rather than continued rapid progression.

These slower expansion rates may reflect attenuated biological activity of the aging aortic wall and reduced abnormal hemodynamic exposure following intervention. Our data, derived from close serial imaging surveillance, confirm that BAV morphology does not inherently accelerate aortic remodeling in this older population. This phenotypic equivalence is strongly supported by real-world data from Bellaire et al. (16), who found no significant differences in aneurysm growth rates or adverse events (dissection, rupture, or death) between conservatively managed BAV and TAV patients. Collectively, these findings reinforce the safety of a watchful waiting strategy for borderline aortic dilatation in older TAVR candidates, which contrasts with classical surgical series in which BAV was associated with more aggressive aortopathy in younger individuals. The absence of aortic events in our cohort further supports this strategy. However, this conservative approach must be combined with strict blood pressure control (target systolic pressure <120 mmHg) and rigorous long-term imaging surveillance. For patients exhibiting mild progression, management remains conservative but includes optimizing antihypertensive therapy and increasing CTA surveillance frequency (e.g., to 6-month intervals).

Notably, although comprised of older adults, our cohort exhibited predominantly low to intermediate surgical risk profiles (median EuroSCORE II <4%). However, the EuroSCORE II primarily estimates mortality for isolated procedures and significantly underestimates the physiological burden of combined aortic valve and ascending aortic replacement (which frequently requires prolonged cardiopulmonary bypass and aortic cross-clamping). Thus, TAVR was selected not because of a high risk for isolated surgery, but because the additive risk of a combined procedure was deemed excessive, or because patients explicitly declined open-heart surgery. Importantly, these encouraging intermediate-term results should not alter current surgical paradigms for fit patients. Consistent with current guidelines, combined open surgery remains our institutional standard of care for operable patients with an aortic diameter >5.0 cm to provide lifetime protection against aortic events. Furthermore, for the extreme subgroup with baseline diameters ≥5.5 cm, TAVR was never a routine choice, but rather a highly individualized “bail-out” alternative reserved solely for patients with absolute surgical contraindications or absolute refusals. Consequently, our findings validate TAVR as a reasonable strategy primarily for older or frail patients for whom combined open surgery is prohibitive. This risk profile may limit generalizability to traditional prohibitive-risk populations.

The principal finding of this study, that BAV and TAV patients exhibited statistically comparable aortic expansion rates following TAVR, warrants careful interpretation within the broader debate surrounding BAV-associated aortopathy. Two prevailing, non-mutually exclusive hypotheses have been proposed to explain ascending aortic dilatation in BAV: the hemodynamic theory and the intrinsic genetic theory. The hemodynamic hypothesis posits that abnormal flow dynamics across a BAV, characterized by eccentric high-velocity jets and asymmetric wall shear stress patterns, drive post-stenotic dilatation of the ascending aorta (5, 17). Conversely, the genetic theory implicates intrinsic structural abnormalities of the aortic wall, including smooth muscle cell apoptosis, elastin fragmentation, and extracellular matrix dysregulation, which predispose the aorta to progressive dilatation independent of hemodynamic loading (18, 19).

Despite BAV patients being younger and having more baseline aortic regurgitation, post-TAVR expansion rates were comparable between the two groups, supporting the hypothesis that abnormal hemodynamics substantially contribute to BAV-associated aortic enlargement, at least in the intermediate term. Notably, these cohorts exhibited distinct baseline drivers of aortic dilation: volume overload from frequent aortic regurgitation in BAV, and pressure overload from prevalent hypertension in TAV. Resolving valvular volume overload via TAVR, alongside strict post-procedural blood pressure control, effectively neutralized these differing mechanical stimuli, likely explaining the comparable aortic stabilization observed in both groups. By alleviating valvular obstruction, TAVR may reduce the mechanical stimulus for further aortic enlargement, which could partly explain the similar post-procedural trajectories observed between the two cohorts. This concept is strongly corroborated by Zheng et al., who demonstrated that although BAV aortas dilated significantly faster than TAV aortas pre-intervention, expansion rates became comparable post-TAVR (20). Although they observed this dynamic in a general AS population, our study extends this physiological concept to older adult patients with pre-existing borderline dilatation (≥4.5 cm). Collectively, these findings suggest that alleviating stenosis may mitigate the excess hemodynamic burden associated with BAV, thereby attenuating, rather than completely abolishing, subsequent aortic enlargement. If intrinsic genetic aortopathy were the sole driver, accelerated BAV dilatation would theoretically persist immediately post-replacement. However, this phenomenon was not observed in our mid-term analysis or in the post-TAVR cohort evaluated by Zheng et al. We acknowledge that our observation window may primarily capture this hemodynamic stabilization, whereas structural gene-mediated medial degeneration may manifest over a substantially longer timeframe.

Following aortic valve intervention, the normalization of transvalvular flow has been shown to substantially reduce these asymmetric wall shear stress patterns, potentially explaining the stabilization of aortic dimensions observed in our cohort (21). However, the median follow-up of 27 months may be insufficient to detect the slow, progressive manifestations of intrinsic aortopathy. Indeed, late aortic complications in surgical BAV series have been reported to emerge predominantly 10–15 years after isolated SAVR, a duration that far exceeds our current observation window (22, 23). Given the relatively low-to-intermediate surgical risk and longer life expectancy of our cohort compared to prohibitive-risk TAVR candidates, achieving intermediate-term aortic stabilization, while encouraging, may not provide definitive lifetime clinical protection. Therefore, the favorable mid-term results reported herein should not be extrapolated to guarantee long-term aortic safety, and extended surveillance remains prudent, particularly for BAV patients.

The overall incidence of moderate or greater paravalvular leakage (PVL) in our cohort was low (2.2%). Moderate or greater PVL occurred exclusively in BAV patients (4.8% vs. 0.0%; *P* = 0.043). This finding likely reflects the anatomical complexity of BAV, including elliptical annuli and heavy calcification (24). However, because the absolute number of patients with moderate or greater PVL was extremely small (*n* = 4), determining its true impact on longitudinal aortic expansion via complex modeling was statistically unfeasible. Therefore, we cannot definitively exclude the possibility that significant residual PVL may accelerate late aortic remodeling (25).

This study has several limitations. First, our retrospective, single-center study is inherently subject to selection bias. Patients with extreme baseline dilatation, rapid historical growth, or high-risk morphological features were likely directed to combined open-heart surgery by the Heart Team. Consequently, the slow aortic expansion rates observed may partially reflect an inherently more benign aortopathy in this selected TAVR cohort. The absence of a surgical comparator group also precludes non-inferiority assessments. Furthermore, due to sample size constraints, we utilized multivariable models rather than propensity score matching to address baseline clinical differences between the BAV and TAV cohorts; thus, a true matched comparison is warranted in future larger studies. Second, the exclusive use of locally manufactured, supra-annular self-expanding valves limits generalizability. Because supra-annular designs generate different flow profiles and wall shear stress compared to intra-annular or balloon-expandable systems, it remains uncertain whether the indolent aortic expansion observed applies equally to patients treated with other platforms or via alternative access routes. Crucially, the utilization of these specific platforms carries an important downstream implication. Should the ascending aorta progress and eventually require surgical replacement, the presence of a supra-annular, self-expanding nitinol frame extending into the sinuses and sinotubular junction materially increases the complexity of that operation. Explantation of these devices carries significant risks of annular and root injury. Furthermore, it frequently converts a potentially straightforward supracoronary graft into a complex root or valve-sparing procedure, and may compel a Bentall or extended reconstruction in patients who were originally deemed unsuitable for combined open surgery. Therefore, deferring aortic intervention is not a “cost-free” decision, as the chosen transcatheter device shapes the difficulty and risk of any future bail-out surgery. This delayed, elevated surgical risk further reinforces the absolute necessity of the strict, long-term imaging surveillance recommended in our study. Third, the median follow-up of 27 months is insufficient to capture very late aortic events. Given the relatively younger age and low-to-intermediate surgical risk of our cohort, these patients possess a longer life expectancy than traditional prohibitive-risk TAVR candidates; thus, extended longitudinal surveillance is imperative to definitively confirm long-term safety. Fourth, the submillimeter expansion rates (0.39–0.43 mm/year) approach the spatial resolution limits of routine CTA, despite our use of standardized ECG-gated imaging and dual-observer measurements. Fifth, while we successfully categorized BAV morphotypes, the small sample sizes within specific subgroups limited our statistical power to evaluate subtype-specific remodeling trajectories. Additionally, detailed anatomical features such as the severity of raphe calcification were not systematically available in our retrospective database, preventing a more granular anatomical risk stratification. Moreover, Sievers Type 0 was the predominant BAV phenotype (55.2%) in our Chinese cohort. Because Type 0 BAV is frequently deferred to surgery in Western practices (where Type 1 prevails), our favorable remodeling results in BAV patients may not be broadly generalizable to international populations. Furthermore, the lack of standardized, longitudinal outpatient blood pressure records and antihypertensive medication logs limits our ability to determine the precise contribution of optimal long-term medical therapy to the observed stabilization of aortic dimensions. Another limitation is that the lack of reliable pre-procedural serial imaging prevents a direct intra-patient comparison of pre- and post-TAVR expansion rates. Finally, the observational nature of the study precludes definitive causal inferences regarding the interplay between hemodynamics, structural integrity, and residual PVL.

## 5 Conclusion

In this retrospective study of 189 patients with severe AS and concomitant ascending aortic dilatation, transfemoral TAVR was associated with favorable aortic outcomes during intermediate follow up, characterized by modest aortic expansion rates and an absence of acute ascending aortic complications. Although the cohort demonstrated slow progressive enlargement of the ascending aorta over time, the rate of progression was low and did not differ significantly between patients with BAV and TAV morphologies. These findings suggest that post-TAVR aortic enlargement is indolent and clinically uneventful during intermediate-term follow-up in properly selected candidates treated with self-expanding platforms, regardless of BAV or TAV morphology. Consequently, in carefully selected older candidates undergoing transfemoral TAVR with self-expanding valves, conservative management of concomitant ascending aortic dilatation may be a reasonable strategy during intermediate-term follow-up, provided that strict long-term blood pressure control and rigorous extended serial imaging surveillance are prioritized and maintained.

## Data Availability

The original contributions presented in the study are included in the article/Supplementary Material, further inquiries can be directed to the corresponding author.
